# Association of ADAM33 gene polymorphisms with adult allergic asthma and rhinitis in a Chinese Han population

**DOI:** 10.1186/1471-2350-9-82

**Published:** 2008-09-09

**Authors:** Dongju Su, Ximei Zhang, Hong Sui, Fuzhen Lü, Lianhong Jin, Jing Zhang

**Affiliations:** 1 Department of Respiratory, The Second Affiliated Hospital of Harbin Medical University, Harbin 150086, PR China; 2 Department of Histology and Embryology, Harbin Medical University, Harbin 150086, PR China; 3 Department of Statistics, Harbin Medical University, Harbin 150086, PR China

## Abstract

**Background:**

Rhinitis and asthma are very common diseases involving genetic and environmental factors. Most patients with asthma also have rhinitis, which suggests the concept of 'one airway, one disease.' A disintegrin and metalloproteinase 33 (ADAM33) is the first asthma-susceptible gene to be discovered by positional cloning. To evaluate the potential influence of ADAM33 gene polymorphisms on allergic rhinitis (AR) and allergic asthma (AS), a case-control study was conducted on the Han population of northeast China.

**Methods:**

Six polymorphic sites (V4, T+1, T2, T1, S1, and Q-1) were genotyped in 128 patients with AR, 181 patients with AS, and 151 healthy controls (CTR). Genotypes were determined by the polymerase chain restriction fragment length polymorphism (PCR-RFLP) method. Data were analyzed using the chi-square test with Haploview software.

**Results:**

The single nucleotide polymorphisms (SNPs), V4 G/C, T+1 A/G, and T1 G/A, of the ADAM33 gene may be the causal variants in AR, whereas ADAM33 V4 G/C, T2 A/G, T1 G/A, and Q-1A/G may participate in the susceptibility of AS.

**Conclusion:**

These results suggest that polymorphisms of the ADAM33 gene may modify individual susceptibility to AR and AS in a Chinese Han population.

## Background

Rhinitis is defined as inflammation of the nasal mucosa and is characterised by nasal discharge, blockage, sneezing, and itching [1,2]. Asthma is a chronic inflammatory disorder of the airway which causes recurrent episodes of wheezing, breathlessness, chest tightness, and coughing in susceptible individuals. These episodes are usually associated with widespread, but variable airflow obstruction that is often reversible, either spontaneously or with treatment. The inflammation also causes an associated increase in existing bronchial hyper-responsiveness (BHR) to a variety of stimuli [1,2]. Epidemiologic studies have consistently shown that asthma and rhinitis often coexist in the same patients in every region of the world [3-5], suggesting the concept of 'one airway, one disease.' The basic premise is that rhinitis and asthma represent the manifestations of one syndrome in two parts of the respiratory tract [6]. In order to raise awareness of the theory of 'one airway, one disease,' the Allergic Rhinitis and Its Impact on Asthma (ARIA) Workshop Group publication proposes three considerations for patients with allergic respiratory disease [7].

ADAM33 is the first reported asthma-susceptible gene identified by positional cloning [8]. ADAM33 is a complex molecule, the expression of which is largely restricted to mesenchymal cells, including fibroblasts and smooth muscle cells, and codes for a protein important for cell fusion, cell adhesion, cell signalling, and proteolysis [9]. The ADAM33 gene is located on chromosome 20p13 and 37 single nucleotide polymorphisms (SNPs) have initially been identified [8]. Recently, several SNPs in the ADAM33 gene have been shown to be significantly associated with asthma, BHR, and atopy [10]. Taken together, these findings suggest an important role for ADAM33 in airway remodelling and asthma [8]. To date, most data available for ADAM33 and asthma have been obtained from Caucasians, but few data on ADAM33 SNPs associated with asthma and atopic disease are available for Asian populations [11-14]. The aim of the present study was to determine whether ADAM33 SNPs are associated with allergic rhinitis (AR) and allergic asthma (AS) in a Chinese Han population.

## Methods

### Subjects

The study group consisted of 128 Chinese patients with AR, 181 patients with AS, and 151 healthy controls (CTR). They were recruited from The 2nd Clinical College of Harbin Medical University in China. The characteristics of the study population are shown in Table 1. The diagnosis of AR was made on the basis of the criteria of the Chinese Society of Allergology (2003). Patients had to satisfy the following AR criteria for inclusion: (1) nasal discharge, blockage, and sneezing, with the 3 symptoms occurring for more than 30 minutes on most days and for more than 6 months per year and (2) persistent symptoms for at least 1 year. AS was diagnosed according to the criteria of the Chinese Society of Allergology (2003). Patients had to satisfy the following AS criteria for inclusion: (1) continual episodes of wheezing and dyspnea which require therapy, (2) clinically-diagnosed wheezing, (3) reversible airway obstruction shown by >15% variation in daily values of volume in the 1^st ^second of forced expiration (FEV1) and peak expiratory flow (PEF) after inhalation or administration of a bronchodilator, and (4) elevated levels of serum total IgE with at least one type of allergen-specific IgE. One hundred fifty-one unrelated healthy Chinese Han adults with no history of asthma and rhinitis were designated as CTR patients. Both patients and the healthy CTR were recruited from the Heilongjiang Province of China between August 2006 and June 2007. The present study was approved by the Ethics Committee of the Harbin Medical University and adhered to the tenets of the Helsinki Declaration. Additionally, the written informed consent was obtained from the patients.

**Table 1:** Description of study population

Characteristic	AR	AS	CTR
Number of patients	128	181	151
Age (years)	39.27 ± 13.53	36.69 ± 11.53	37.18 ± 10.60
Gender (Female)	44(34.38%)	67(37.02%)	54(35.76%)
FEV1 (%)	100.95 ± 5.36	73.38 ± 10.66	102.84 ± 3.11

### DNA extraction and genotyping

Genomic DNA was extracted from 5 ml of frozen whole blood using a DNA Extraction Kit (Qiagen, Germany), according to the manufacturer's protocol and following the manufacturers' instructions. We selected 6 SNPs in the ADAM33 gene, which previously had been shown to have an allelic and/or haplotype association with AS, including V4(rs2787094), T+1(rs2280089), T2(rs2280090), T1(rs2280091), S1(rs3918396), and Q-1(rs612709). Using the unique rs accession numbers, SNP details and sequence data are available through NCBI databases . The polymorphic region was amplified by PCR with a TGradient Thermoblock PCR System (Iometra, Germany) in a 25 μl reaction solution containing 0.3 μg genomic DNA, 1× PCR buffer, 0.3 mM MgCl_2_, 0.2 mM dNTPs, 2 U TaqDNA polymerase (Takara, Japan), and 0.1 μmol of each primer (Invitrogen, USA). Genotyping primers and PCR programs are shown in Table 2. PCR products were digested overnight with restriction enzymes (NEB, UK), according to the manufacturer's protocol, and analyzed by 2% agarose gel electrophoresis. The restriction enzymes and the length of the digested fragments are shown in Table 3.

**Table 2:** Primers and PCR programs for ADAM33 PCR-RFLP genotyping

Reference SNP ID	Gene/SNP primer	Sequence	PCR program
rs2787094	ADAM33 V4C/G F	5'-ACACACAGAATGGGGGAGAG-3'	94°C 5 min; 35 cycles, 94°C 30 s,
	ADAM33 V4C/G R	5'-CCAGAAGCAAAGGTCACACA-3'	53°C 30 s, 72°C 30 s; 72°C 5 min
rs2280089	ADAM33T+1A/G F	5'-CTGAGCCCAGAAACCTGATT-3'	94°C 5 min; 35 cycles, 94°C 30 s,
	ADAM33T+1A/G R	5'-AGAAGGGAAGGGCTCATGC-3'	54°C 30 s, 72°C 30 s; 72°C 5 min
rs2280090	ADAM33 T2A/G F	5'-TTCTCAGGGTCTGGGAGAAA-3'	94°C 5 min; 35 cycles, 94°C 30 s,
	ADAM33 T2A/G R	5'-GCCAACCTCCTGGACTCTTA-3'	52°C 30 s, 72°C 30 s; 72°C 5 min
rs2280091	ADAM33 T1A/G F	5'-ACTCAAGGTGACTGGGTGCT-3'	94°C 5 min; 35 cycles, 94°C 30 s,
	ADAM33 T1A/G R	5'-GAGGGCATGAGGCTCACTTG-3'	54°C 30 s, 72°C 30 s; 72°C 5 min
rs3918396	ADAM33 S1A/G F	5'-TGTGCAGGCTGAAAGTATGC-3'	94°C 5 min; 35 cycles, 94°C 30 s,
	ADAM33 S1A/G R	5'-AGAGCTCTGAGGAGGGGAAC-3'	50°C 30 s, 72°C 30 s; 72°C 5 min
rs612709	ADAM33 Q-1A/G F	5'-GGATTCAAACGGCAAGGAG-3'	94°C 5 min; 35 cycles, 94°C 30 s,
	ADAM33 Q-1A/G R	5'-GTTCACCTAGATGGCCAGGA-3'	60°C 30 s, 72°C 30 s; 72°C 5 min

**Table 3:** Restriction enzymes and length of digested fragments

	V4C/G	T+1A/G	T2A/G	T1A/G	S1A/G	Q-1A/G
Enzyme	PstI	HpyAV	HpyCH4III	NcoI	HinfI	BtsCI
Length of digested	G: 168+206	A: 284+28	A: 198+112	A: 140+260	G: 132+172	A: 20+138
fragments (bp)	C: 374	G: 312	G: 310	G: 400	A: 304	G: 158

### Statistical analyses

We tested for the Hardy-Weinberg equilibrium (HWE) among cases and controls separately. The genotype and allele frequencies were obtained by direct counting. Statistical significance was defined as a P < 0.05. In order to obtain a measure of significance corrected for multiple testing bias, we ran 10,000 permutations to compute P-values using the Haploview program. Comparisons of the distributions of the allele and genotype frequencies were performed using the chi-square test. The relative risk associated with rare alleles was estimated as an odds ratio (OR) with a 95% confidence interval (CI).

## Results

The genotype and allele frequencies of the ADAM33 gene polymorphisms in patients and healthy controls are shown in Tables 4, 5 and 6. All of the six SNPs genotyped were in HWE (P > 0.05) in the case and control cohorts. The ADAM33 V4 G, T+1 A, and T1 G allele carrier frequencies were significantly higher in AR patients than in the CTR patients (P < 0.0001, = 0.0037, and < 0.0001, respectively). Statistical analysis revealed no significant differences in the allele frequencies in T2 A/G, S1 G/A, and Q-1 A/G between AR and CTR patients. The differences at the V4, T2, T1, and Q-1 sites between the AS and CTR groups were significant (P < 0.0001). In contrast, no significant differences in the allele frequency distribution at the T+1 and S1 sites between the AS and CTR groups were observed. The ADAM33 V4 G, T2 A, T1 G, and Q-1 A allele frequencies were significantly higher in the AS patients than in the CTR patients (P < 0.0001). Correction for multiple tests was performed in this study.

**Table 4:** Genotype frequency distribution and association study in ADAM33 between AR and CTR

Genotype		Frequency, no.(%)		x^2 ^P value	Odds ratio
				
		AR(n = 128)	CTR(n = 151)		(95% CI)
rs2787094	CC	46(35.94)	113(74.84)	<0.0001	0.19(0.11–0.32)
	CG	60(46.88)	32(21.19)	<0.0001	3.28(1.95–5.53)
	GG	22(17.18)	6(3.97)	0.0003	5.02(1.97–12.80)
rs2290089	AA	4(3.13)	0	0.0432	
	AG	51(39.84)	41(27.15)	0.0246	1.78(1.07–2.94)
	GG	73(57.03)	110(72.85)	0.0056	0.49(0.30–0.82)
rs2280090	AA	0	0		
	AG	24(18.75)	12(7.95)	0.0073	2.67(1.28–5.59)
	GG	104(81.25)	139(92.05)	0.0073	0.37(0.18–0.78)
rs2280091	AA	56(43.75)	117(77.48)	<0.0001	0.23(0.13–0.38)
	AG	55(42.97)	29(19.21)	<0.0001	3.17(1.86–5.41)
	GG	17(13.28)	5(3.31)	0.0021	4.47(1.60–12.49)
rs3918396	AA	100(78.12)	110(72.85)	0.3086	
	AG	28(21.88)	41(27.15)	0.3086	
	GG	0	0		
rs612709	AA	0	0		
	AG	16(12.5)	11(7.28)	0.142	
	GG	112(87.5)	140(92.72)	0.142	

**Table 5:** Genotype frequency distribution and association study in ADAM33 between AS and CTR

Genotype		Frequency, no.(%)		x^2 ^P value	Odds ratio
				
		AS(n = 181)	CTR(n = 151)		(95% CI)
rs2787094	CC	49(27.07)	113(74.84)	<0.0001	0.12(0.08–0.20)
	CG	78(43.09)	32(21.19)	<0.0001	2.82(1.73–4.59)
	GG	54(29.84)	6(3.97)	<0.0001	10.28(4.28–24.69)
rs2290089	AA	0	0		
	AG	37(20.44)	41(27.15)	0.151	
	GG	144(79.56)	110(72.85)	0.151	
rs2280090	AA	4(2.21)	0	0.1289	
	AG	49(27.07)	12(7.95)	<0.0001	4.30(2.19–8.44)
	GG	128(70.72)	139(92.05)	<0.0001	0.21(0.11–0.41)
rs2280091	AA	63(34.81)	117(77.48)	<0.0001	0.16(0.10–0.25)
	AG	78(43.09)	29(19.21)	<0.0001	3.19(1.93–5.26)
	GG	40(22.10)	5(3.31)	<0.0001	8.28(3.18–21.59)
rs3918396	AA	140(77.35)	110(72.85)	0.3437	
	AG	41(22.65)	41(27.15)	0.3437	
	GG	0	0		
rs612709	AA	0	0		
	AG	44(24.31)	11(7.28)	<0.0001	4.09(2.03–8.24)
	GG	137(75.69)	140(92.72)	<0.0001	0.24(0.12–0.49)

**Table 6:** Allele frequency distribution and association study in ADAM33

Name	SNP	Minor allele frequency			P-value		Permutation p-value		Odds ratio (95% CI)	
		
		AR (n = 128)	AS (n = 181)	CTR (n = 151)	AR vs CTR	AS vs CTR	AR vs CTR	AS vs CTR	AR vs CTR	AS vs CTR
Rs2787094	G/C	0.406	0.514	0.146	<0.0001	<0.0001	<0.0001	<0.0001	0.25(0.17–0.37)	0.16(0.11–0.24)
rs2280089	A/G	0.231	0.102	0.136	0.0037	0.1812	0.0279		1.91(1.23–2.96)	
rs2280090	A/G	0.094	0.158	0.04	0.0097	<0.0001		<0.0001		4.52(2.37–8.59)
rs2280091	G/A	0.348	0.437	0.129	<0.0001	<0.0001	<0.0001	<0.0001	0.28(0.18–0.42)	0.19(0.13–0.28)
rs3918396	G/A	0.109	0.113	0.136	0.3454	0.3802				
rs612709	A/G	0.063	0.121	0.036	0.1526	<0.0001		0.0016		3.66(1.86–7.22)

## Discussion

The work by van Eerdewegh et al. [8] that identified ADAM33 as an asthma-susceptible gene using positional cloning and an association study had a profound impact on researchers investigating the genetics of bronchial asthma. Polymorphisms of ADAM33 were originally shown to be associated with BHR. In previous studies, T1, T2, S2, and V-3 sites were reported in Japanese adult populations [13,15], and these associations were also found in our samples. In contrast, no association was found between asthma and ADAM33 in Puerto Rican and Mexican populations [16], and a Korean population [12]. The study by van Eerdewegh [8] was the first to show that ADAM33 polymorphisms (Q-1, S1, ST+4, ST+7, V-1, and V4) and haplotypes were associated with asthma in Caucasian families. Recently, Raby et al. [17] performed a family-based association study for childhood asthma and ADAM33 SNPs in 652 nuclear families. They showed that no single SNP was associated with childhood asthma in Caucasians, but that a common haplotype of ADAM33 was associated with the disease. They also reported that T1 and T+1 were marginally associated with childhood asthma in Hispanics. In previous association studies of ADAM33 and asthma/asthma-associated phenotypes, no single ADAM33 SNP was consistently associated with asthma or intermediate phenotypes. This may be because of allelic heterogeneity, a single disorder caused by different mutations within a gene [14]. A number of studies have been conducted to replicate the original findings, some confirming and others refuting the association of ADAM33 with asthma [17-19]. In the present study, adult asthma was associated with ADAM33 SNPs, V4, T2, T1, and Q-1, but not with the T+1 and S1, and the V4, T+1, and T1 sites were also found to have significant associations with AR after correction for multiple tests. Comparison of our results with those studies indicates that no single SNP was universally associated with the asthma phenotype. The genetic basis of asthma may differ between different ethnic groups; it is possible that a particular subset of SNPs may be a risk factor for asthma in Caucasians, and that a different subset may increase the risk in Asian populations [13].

Epidemiological studies have clearly shown that rhinitis and asthma are frequently concurrent [20,21]. The majority of patients with AS also have AR, which presents in more than 75% of the patients with allergic asthma [22]. The relationship between AR and AS has become a focus of research. Recent advances have demonstrated that systemic inflammatory responses play a critical and integrating role in the pathophysiology of airway disease. Substantial evidence has also shown that AR and AS are closely linked, and the concept of 'one airway, one disease' has emerged [23]. Under this hypothesis, AS and AR are regarded as a combined entity of upper and lower airway disease influenced by the inflammatory process. An alteration of upper airway function could therefore affect the function of the lower airway. Improvement of AR symptoms is associated with resolution of AS symptoms. On the other hand, deterioration of AR symptoms may exacerbate AS symptoms. Consequently, appropriate management of AR could decrease the risk of AS developing or AS exacerbation [23]. The results of several epidemiologic surveys presented at the European Respiratory Society Annual Congress (ERS) in 2007 supported the concept of 'one airway, one disease' [24,25]. In this context, we genotyped ADAM33 variants in AR cases and healthy individuals to evaluate the potential influences of ADAM33 gene polymorphisms on AR risk, and to determine whether ADAM33 is also the susceptible gene for AR. As expected, some significant associations were observed in AR patients. Although there are some differences between AR and AS in the statistical analysis results with respect to ADAM33 genetic susceptibility, polymorphisms of the ADAM33 gene may modify individual susceptibility to AR and AS in a Chinese Han population.

## Conclusion

Our data suggest that the ADAM33 gene may be involved in the susceptibility to AR and AS in a Chinese Han population. The SNPs (V4 G/C, T+1 A/G, and T1 G/A) of the ADAM33 gene may play an important role in the susceptibility of AR disease, whereas ADAM33 V4 G/C, T2 A/G, T1 G/A, and Q-1A/G may participate in the susceptibility to AS. Considering that the limited sample size may produce relative risk estimates lacking adequate precision, extended analyses with a larger sample size should be carried out from different ethnic origins to further verify this association.

## Competing interests

The authors declare that they have no competing interests.

## Authors' contributions

D–J S performed the primer design. X–M ZH performed the PCR-RFLP experiments and wrote the drafts. HS contributed statistical analysis. F–ZH L and L–H J conceived of the study, and participated in its design and coordination and helped to draft the manuscript. J–ZH collected the patient and control blood samples. All authors read and approved the final manuscript.

## Pre-publication history

The pre-publication history for this paper can be accessed here:


